# Analysis of causes of death among HIV-infected patients of Kiev Regional AIDS Center during 2013

**DOI:** 10.7448/IAS.17.4.19617

**Published:** 2014-11-02

**Authors:** Tetiana Stepchenkova, Olena Martynenko, Oleksandr Yurchenko

**Affiliations:** 1 Virological, National Medical Academy of Post-graduate Education, Kiev, Ukraine; 2 Ambulatory, Kiev Regional AIDS Center, Kiev, Ukraine; 3 Kiev Regional AIDS Center, Kiev, Ukraine

## Abstract

Ukraine is a leader in Europe in the prevalence of HIV infection. There are up to 270 thousand people, who are living with HIV. Since 1987, in Ukraine, 33,149 HIV-infected people died. During the first six months of 2013, of all the dead, who were suffering from HIV and in need of antiretroviral treatment (ART) at the time of death, 41% received treatment and only 5.7% received ART for more than one year. Specialists of Kiev Regional AIDS Center analyzed mortality among the patients of the centre, in order to determine the most frequent cause of death, set the group most at risk and to develop effective measures to reduce mortality among HIV-infected patients. In Kiev AIDS Center, 10,000 people are under medical observation and 4004 of them are taking ART. During 2013, 305 persons died: 217 were women and 88 were men which included 3 children under 14 years. Most of the dead – 272 (89%) were aged 25–49. Among the total number of the dead, 125 people (41%) were receiving ART, 53 of them (17%) were receiving ART for at least one year and 39 of them (13%) were receiving ART for less than one month. Hundred and fifty-eight people (52%) required ART and 22 (7%) did not need therapy. Hundred and ninety-two patients (63%) were in four clinical stage of HIV infection. Hundred and ten of them had HIV+TB co-infection. Twenty patients died due to TB and 12 patients died due to hepatitis b virus/hepatitis c virus (HBV/HCV). Among these patients, 87 people (39%) were taking ART and 136 persons (61%) were in need of ART, but did not get it. Nineteen patients were diagnosed with cancer. Sixteen patients, who were co-infected HIV+TB had a CD4 cell count of more than 300. Based on this analysis, we can conclude that the main causes of high mortality among HIV-infected patients in 2013 were late diagnosis of HIV, besides a large number (52%) of patients, who were in need of ART did not take it. A large number (40%) among those who died were patients co-infected with HIV+TB, HIV+HBV/HCV. This reinforces the necessity to introduce new schemes for the integrated treatment of these patients, including patients with MDR, XDR TB. Also all HIV-infected patients who died in 2013, were diagnosed multiple pathology of various organs, which demonstrates the necessity of the introduction of schemes of early diagnosis and approaches to treatment of this disease. A large number of deaths (24%) of patients receiving ART for more than a year, highlight the necessity for changes to existing treatment regimen in HIV patients, with the possibility of the development of viral resistance to certain drugs and taking into account the latest international experience in the treatment of HIV-infected patient.

